# Alkaptonuria: Clinical Spectrum of a Diagnosed Case in Bahrain With a Literature Review

**DOI:** 10.7759/cureus.77174

**Published:** 2025-01-09

**Authors:** Zahra Alsahlawi, Layla I Salman, Amina M Alaradi, Fatema Hamada, Huda S Alsahlawi, Zahra M Ali

**Affiliations:** 1 Pediatrics, Salmaniya Medical Complex, Manama, BHR; 2 Pediatrics, Arabian Gulf University, Manama, BHR; 3 Radiology, Salmaniya Medical Complex, Manama, BHR; 4 Rheumatology, Salmaniya Medical Complex, Manama, BHR

**Keywords:** bahrain, cartilage damage, nitisinone, tyrosine, alkaptonuria

## Abstract

Alkaptonuria (AKU) is a rare metabolic condition caused by mutations within a gene coding for homogentisate 1,2 dioxygenase enzyme involved in the tyrosine catabolism pathway. This mutation will result in the accumulation of homogentisic acid (HGA) in the body. AKU is a multi-systemic slowly progressing disease. The onset of its clinical presentation may vary based on the extensive disposition of the HGA. Initially, it might be asymptomatic, and symptoms usually appear in the second or third decades due to the formation of HGA, melanin compounds that accumulate in the cartilage leading to ochronosis. Furthermore, by the fourth or fifth decade, ochronotic arthropathy occurs, along with other extra-articular complications such as cardiovascular manifestations (e.g., valvular heart disease), renal and prostatic stones, and hypothyroidism. Management of this condition is mainly symptomatic, focusing on the treatment of its complications. Recently, the use of nitisinone (NTBC) has shown stabilization of disease manifestations. In this report, we present in detail the first AKU-diagnosed patient, including the clinical presentations, radiological findings, genetic results, and clinical outcomes, from the main tertiary hospital in Bahrain. Moreover, we conducted a thorough literature review on this rare condition.

## Introduction

Alkaptonuria (AKU; Online Mendelian Inheritance in Man (OMIM): 203500) is a rare genetic autosomal recessive condition caused by mutations within a gene coding for homogentisate 1,2 dioxygenase (HGD, Enzyme Commission (EC): 1.13.11.5) that plays a role in the tyrosine catabolism pathway by converting homogentisic acid (HGA) into maleylacetoacetic acid; this mutation will result in accumulation of HGA in the body. The prevalence of AKU is estimated at approximately 1:250,000 to 1:1,000,000 worldwide with higher incidences in Slovakia, Dominican Republic, India, and Jordan [1-3]. The incidence of affected males and females was represented equally, although males had higher morbidity [1]. AKU is a multi-systemic, slowly progressing disease [4]. The onset of its clinical presentation may vary based on the extensive disposition of the HGA. Initially it might be asymptomatic [5]. However, it can be easily diagnosed in childhood through the darkening of urine on standing, which is considered the earliest pathognomonic sign of the disease [3,6]. The symptoms usually appear in the second or third decade due to the formation of HGA, melanin compounds that accumulate in the cartilage, leading to ochronosis [5]. Furthermore, by the fourth or fifth decade, ochronotic arthropathy occurs, along with other extra-articular complications, such as cardiovascular manifestations like valvular heart disease, renal and prostatic stones, and hypothyroidism [3]. When the disease is suspected, the diagnosis can be confirmed by gas chromatography-mass spectrometry (GC-MS) through which a significant amount of HGA will be identified in the urine [1,3]. The usual amount of HGA excreted per day in AKU patients is around 1-8 g [1]. In addition, molecular genetic testing can be done to detect different mutations in the HGD gene. There is no curative treatment for AKU in which supportive treatment is used to maintain quality of life. The clinical trials conducted between 2014 and 2020 demonstrated that the use of nitisinone (NTBC) has a significant effect on reducing the manifestations of the disease by inhibiting the production of HGA. Meanwhile, it increases plasma tyrosine levels, so a low-protein diet is recommended [7,8]. The total reported number of AKU cases worldwide is around 1233, according to an article by Zatkova et al. published in 2020 [8]. Fifteen additional cases have been reported between 2021 and 2023. A limited number of studies have been conducted in the Gulf region, revealing about eight AKU patients. In this study, we aim to discuss the first AKU-diagnosed patient in Bahrain.

## Case presentation

A 41-year-old Bahraini male, born to non-consanguineous parents, a Bahraini mother and an Egyptian father, smokes and has a body mass index (BMI) of 39 kg/m². He is known to have hypertension and dyslipidemia, both controlled by Triplixam 10 mg/2.5 mg/5 mg and atorvastatin 20 mg, respectively. He is also taking alprazolam 15 mg and escitalopram 20 mg for depression management.

**Figure 1:**
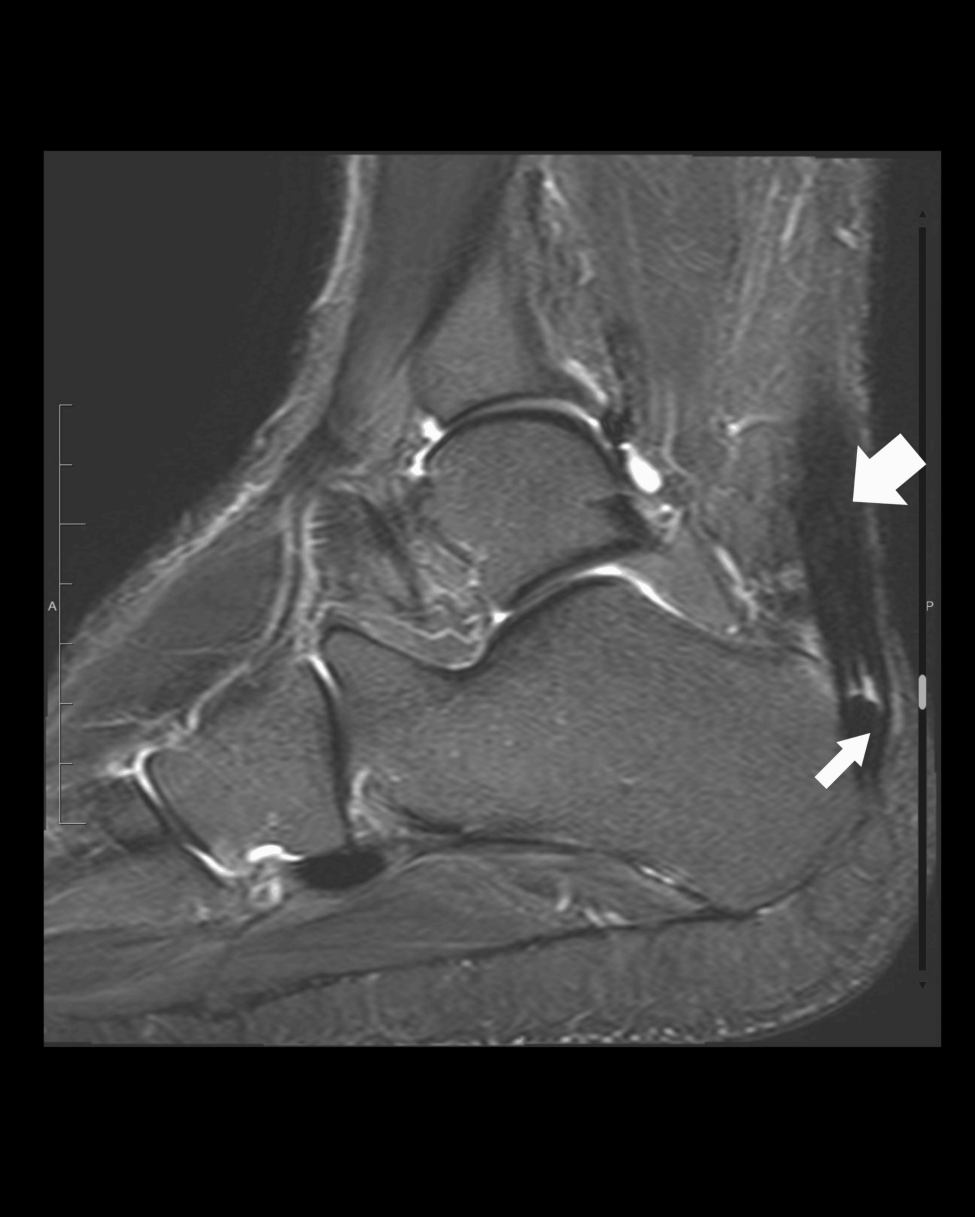
Radiological findings at patient presentation. Left ankle MRI sagittal STIR image showing chronic tendinopathy (bold arrow) with a partial tear of the Achilles tendon near the calcaneal insertion (thin arrow).

**Figure 2:**
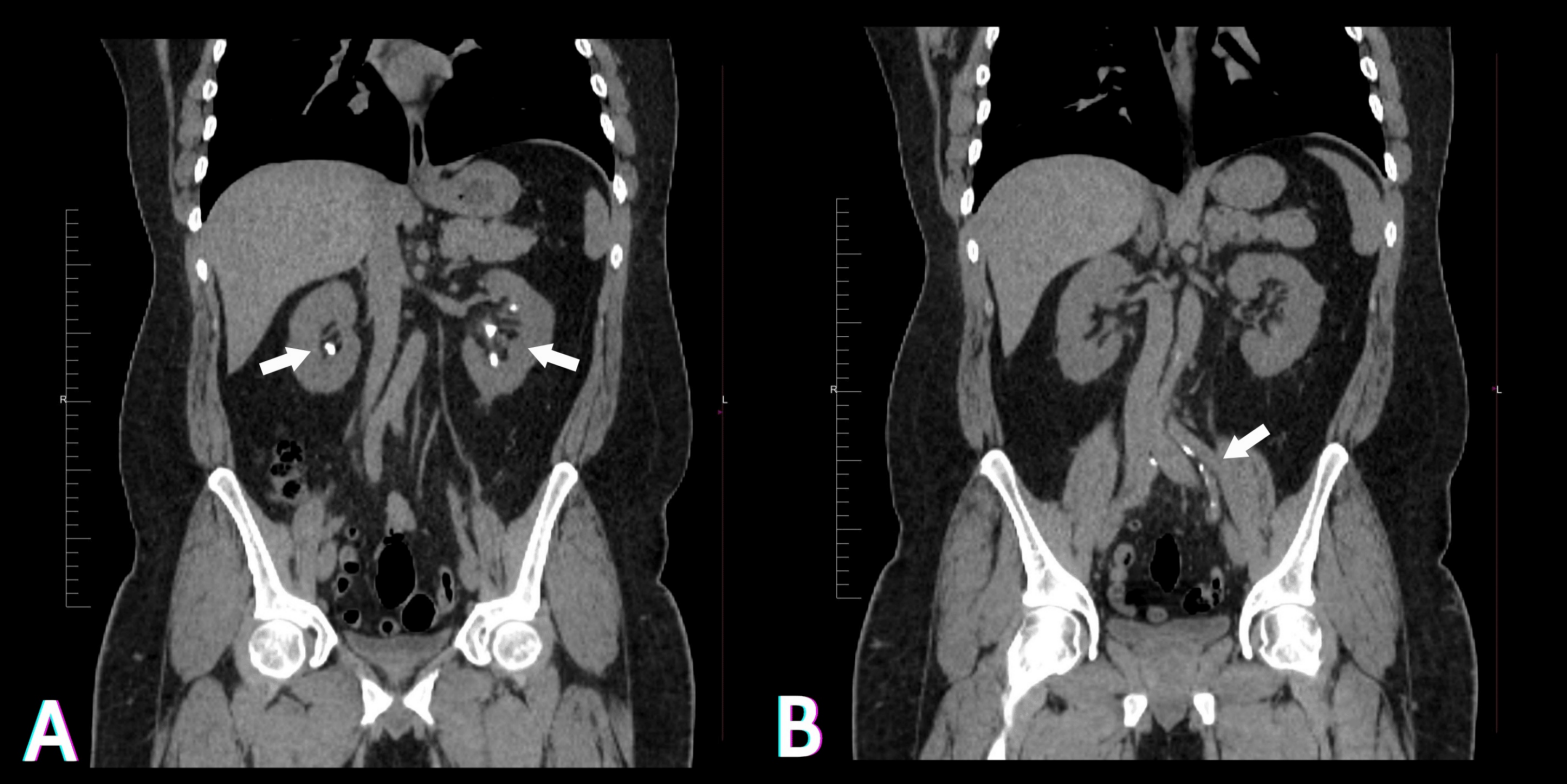
Coronal non-enhanced CT scan of the abdomen and pelvis. (A) Multiple bilateral non-obstructive renal calculi; (B) calcifications in the left common iliac artery.

At the age of 37, he presented to the hospital with a history of a click heard over the left ankle while exercising, associated with painful swelling and an inability to walk. Examination revealed a positive Thompson test, which suggested an Achilles tendon rupture. An MRI of the ankle (Figure 1) was performed to rule out any other possible injuries, followed by admission and tendon repair. Upon presentation, the patient reported a history of multiple non-obstructive urinary stones, which began developing during his adolescence, as shown in a non-enhanced CT scan of the abdomen and pelvis (Figure 2). He had undergone frequent extracorporeal shock wave lithotripsy (ESWL) for these stones. Incidentally, calcifications were detected in both common iliac arteries on the CT scan, more significantly in the left common iliac artery (Figure 2).

**Figure 3:**
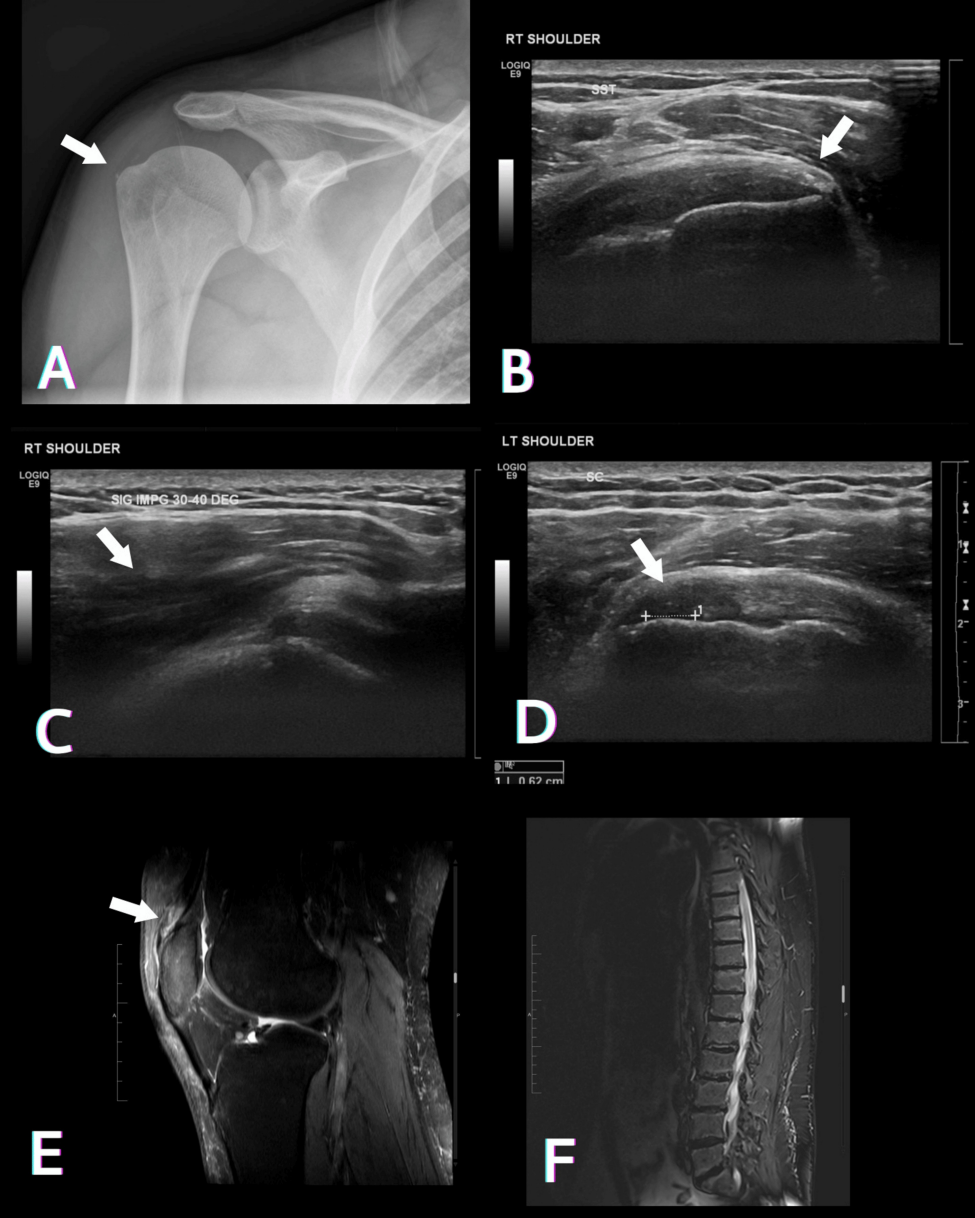
Radiological findings of several presentations of multiple joint pain. (A) Plain X-ray of the right shoulder showing calcifications at the insertion of the supraspinatus tendon. (B) Right shoulder ultrasonography showing calcifications at the insertion of the supraspinatus tendon. (C) Right shoulder ultrasonography showing significant limitation of abduction and positive subacromial impingement, with limited abduction at 30-40 degrees. (D) Left shoulder ultrasonography showing a stable partial thickness tear of the subscapularis tendon. (E) Left knee MRI sagittal PD FS showing quadriceps tendinopathy and partial tear. (F) Sagittal view of thoracolumbar spine MRI STIR showing multilevel degenerative changes of the lower thoracic and lumbar spine, including end plate changes and intervertebral disc degenerative changes.

Over the following months, he had several episodes of multiple joint pain that interfered with his work, resulting in early retirement. Various imaging modalities were performed, but a provisional diagnosis was not reached. The pain affected his shoulders, knees, and lower back (Figure 3).

Due to the patient’s age, clinical manifestations, and radiological findings, he was referred to a rheumatology clinic to rule out possible rheumatological causes. Initially ankylosing spondylitis was suspected, and he was planned to receive Humira. MRI showed multi-level degenerative changes without the presence of a specific diagnosis (Figure 3). However, ankylosing spondylitis was less likely. Therefore, the exclusion of systemic diseases had to be done. The patient was thoroughly investigated to rule out differential causes like calcium pyrophosphate deposition disease, gout, hemochromatosis, and AKU. Seronegative spondyloarthropathy markers, including human leukocyte antigen-B27 and antinuclear antibody, were negative, and other rheumatological workups were normal. The diagnosis of AKU was made based on the high level of urine HGA, as shown in Table 1.

**Table 1:** Laboratory investigations of the patient. The table showed a significantly high level of urine HGA with normal values of the rheumatological workup done during his presentation.

Parameters	Patient values	Reference range
Rheumatoid factor	9.19 IU/mL	≤ 14 IU/mL
Anti-cyclic citrullinated peptide antibody	0.5 U/mL	≤ 10 U/mL
Uric acid	466 μmol/L	220-547 μmol/L
Complement 3	169 mg/dL	90-180 mg/dL
Complement 4	19.7 mg/dL	10-40 mg/dL
Urine HGA	5.5 g/L	≤ 0.1 g/L

Following this, he was referred to a metabolic clinic for further workup. A more detailed history revealed the presence of dark urine in diapers since birth, which had been ignored, as well as conjunctival pigmented lesions, blurry vision, eye floaters, bluish discoloration of the digits and toes, and right ear pigmentation.

Based on the patient’s history and positive HGA, genetic testing was performed, which confirmed the diagnosis of AKU. It revealed compound heterozygous variants in the HGD gene. A heterozygous 2-base pair deletion in exon 6 of the HGD gene (chr3:g.120650831_120650832del; Depth: 171x) was detected, resulting in a frameshift and premature truncation of the protein 2 amino acids downstream of codon 126 (p.Lys126ValfsTer2; ENST00000283871.10). The observed variation has previously been reported in patients affected with AKU [9,10]. The variant has not been reported in the 1000 genomes, Genome Aggregation Database (gnomAD), and our internal databases. The in-silico prediction of the variant indicates it is damaging according to MutationTaster2. The reference region is conserved across species. Based on the above evidence, this HGD variation is classified as a pathogenic variant.

A heterozygous missense variation in exon 3 of the HGD gene (chr3:g.120674937G>A; Depth: 150x) that results in the amino acid substitution of leucine for serine at codon 47 (p.Ser47Leu; ENST00000283871.10) was detected. It lies in the homogentisate 1,2-dioxygenase domain of the HGD protein [11]. The observed variation has previously been reported in patients affected with AKU [12]. This variant has not been reported in the gnomAD databases and has a minor allele frequency of 0.02% and 0.001% in the 1000 genomes and our internal databases, respectively. The in-silico predictions of the variant suggest it is possibly damaging according to PolyPhen-2 (HumDiv) and damaging according to SIFT, LRT, and MutationTaster2. The reference codon is conserved across species.

Family screening was arranged; a similar disease mutation was detected in two of his younger sisters, while his third sister had a normal genetic test. He is married to a healthy woman, and his two daughters were heterozygous for the disease. The patient was followed in the metabolic clinic regularly and NTBC 10 mg daily was prescribed for him. He has been compliant with NTBC for more than 18 months to date with no further tendon or joint complaints. Recently, the patient suffered an acute non-ST-elevation myocardial infarction. Following this, he was admitted to the cardiac care unit and started on bisoprolol 2.5 mg, atorvastatin 40 mg, clopidogrel 75 mg, aspirin 81 mg, and fondaparinux 2.5 mg. His troponin level on admission was 0.51 ng/mL (normal ≤1.5 ng/mL) and rose to 20 ng/mL within the following hours. Initial electrocardiograms (ECGs) were normal, and the echocardiogram (ECHO) showed an ejection fraction of 75% along with evidence of hypertensive heart disease and ischemic heart disease. As a result, he underwent cardiac catheterization.

## Discussion

AKU is a rare hereditary autosomal recessive metabolic disorder caused by pathogenic variants in the HGD gene, resulting in HGD enzyme deficiency and HGA accumulation. The consequent elevation of HGA level is either excreted in urine or oxidized via a benzoquinone acetic acid to form a melanin-like pigment, which deposits in connective tissues in a process known as ochronosis. Ultimately the ochronotic tissue becomes fragile, stiff, and susceptible to chronic inflammation.

The worldwide incidence of AKU is estimated around 1:250,000 to 1:1,000,000 although it may be underestimated in view of the subtle nature of the disease. Consanguinity plays a role in increasing the incidence of AKU in the Middle East region. However, the late diagnosis in our case could be attributed to the non-consanguineous parents in addition to the lack of awareness about the disease in our region. The total reported number of AKU cases worldwide is around 1233 according to Zatkova et al. article, which was published in 2020 [8]; 15 additional cases were reported between 2021 and 2023. A limited number of studies have been conducted in the Gulf region, revealing approximately eight AKU patients. Our patient is the first reported AKU case in Bahrain.

Darkening of urine on standing is considered as the earliest clinical manifestation of the disease and it is the only presenting feature during infancy period. Yet, most clinical manifestations present after the third decade of life, mainly affecting the musculoskeletal, renal, and cardiovascular systems as well as the sclera and ears. The severity of the disease varies among individuals with males being more severely affected than females.

The renal system is the first to be affected by AKU. It manifests at birth with urine that turns dark brown upon standing, a change that occurs several hours after voiding. Therefore, it usually goes unnoticed by the parents or the patient. When noticed, it could be the pathognomonic sign of the disease, and the diagnosis can be confirmed by GS-MS analysis. Moreover, late renal presentations may include lithiasis, calcifications, and irreversible decline of renal function. It has also been reported that patients with AKU developed prostate calcification at younger age [1]. Our patient provided a history of black urine in diapers, which was initially ignored. During adolescence, he developed renal stones, which were managed with multiple sessions of ESWL, but no obvious underlying cause was identified at that time.

Meanwhile, due to reduced renal clearance of HGA with age, musculoskeletal manifestations are typically delayed until the third or fourth decade of life [13]. These manifestations are divided into three main categories: early onset arthropathy, tendinopathy, and osteopenia/osteoporosis [13]. Data indicates that 95.6% of AKU patients experienced joint pain according to Rudebeck et al. [4]. Patients mostly present with back stiffness, with eventual loss of lordosis and exaggeration of thoracic kyphosis as the earliest involved joint is the spine [13].

Due to the similarity in symptoms between AKU and ankylosing spondylitis, AKU patients are often misdiagnosed, as in our case. The radiological sparing of the sacroiliac joints and the absence of a bamboo spine can help point toward a diagnosis of AKU [13].

The most affected peripheral joint is the knee, which represents 64% of cases [13]. Thus, AKU patients can be misdiagnosed with osteoarthritis. Therefore, synovial fluid aspiration is used to distinguish between both conditions, as ochronotic arthritis will reveal floating black particles, also called ground pepper signs [13].

Moreover, spontaneous tendon or ligament ruptures are estimated to occur in approximately 20-30% of cases [13]. The most commonly affected tendons are the patellar and Achilles tendons [13]. Suspicion and diagnosis can often be confirmed intraoperatively upon direct visualization of the bluish-black ochronotic tissue [5,13,14]. Our patient had recurrent tendon ruptures and underwent surgical repairs.; unfortunately, due to the rarity of the disease and the lack of awareness, this discoloration was neglected, and the diagnosis was missed.

In addition, AKU patients can present to otolaryngologists or ophthalmologists complaining of ears or sclera hyperpigmentation. Furthermore, cardiovascular involvement has been reported in AKU patients as a consequence of ochronotic pigment deposition within the heart valves, endocardium, and coronary vessels [5]. Also, he suffered from an acute myocardial infarction although the cause could be attributed to his medical co-morbidities.

The gold standard method of diagnosis is the GC-MS, which detects the presence of HGA in urine. Sanger sequencing is also essential to screen and identify the variant mutations of the disease among family members.

To date, there is no curative treatment for AKU. However, supportive management including dietary modifications, ascorbic acid, painkillers, and physiotherapy can improve the quality of life. Since HGA buildup is a result of phenylalanine and tyrosine metabolism, a dietary restriction of food containing high levels of these amino acids such as meat, poultry, seafood, eggs, dairy products, and nuts is recommended. Ascorbic acid (vitamin C) acts as an antioxidant, which promotes HGA, phenylalanine, and tyrosine excretion in urine [5]. However, its clinical effect is highly debatable and needs further evaluation. Joint replacement surgery is reserved for patients with spinal or peripheral large joint involvement to improve the quality of survival. NTBC is a US Food and Drug Administration-approved treatment for hereditary tyrosinemia type 1. It also plays a role in lowering HGA levels by inhibiting hydroxyphenyl-pyruvate dioxygenase enzyme. Ranganath et al. [15] published a study entitled Suitability of NTBC in AKU 1 (SONIA1) investigating the effect of different doses of NTBC once daily on 24-hour urinary HGA excretion in AKU patients after four weeks of treatment. They concluded that daily 8 mg of NTBC is the most efficacious dose that corresponded to a mean reduction in 24-hour urinary HGA excretion by 98.8% compared with baseline. Ranganath et al. [16] continued with SONIA2, which aimed to evaluate the efficacy and safety of 10 mg NTBC once daily over four years. They concluded that NTBC 10 mg daily was effective in reducing urine and serum HGA; it also showed that NTBC reversed the ochronotic process and improved clinical signs, which helped in slowing the disease progression. The patient in our case report showed stabilization of disease manifestations after starting NTBC. On the other hand, NTBC administration results in elevation of serum tyrosine level, which might cause ocular manifestations including keratopathy, diminished visual acuity, and corneal tissue damage. Regular slit-lamp examination is necessary to detect keratopathy in NTBC-treated AKU patients, as it can be silent. However, ocular symptoms are reversible after at least two months of NTBC withdrawal. Moreover, high tyrosine levels can lead to brain dysfunction and cognitive impairment as it can cross the blood-brain barrier [17].

## Conclusions

The rarity of AKU, along with the lack of local knowledge and expertise, makes the diagnosis difficult to diagnose. In our case, although his presentation was early, occurring in infancy and early adolescence, his diagnosis was delayed until his late thirties. Early administration of NTBC along with a low protein diet can delay the disease progression and improve the long-term outcome. However, careful monitoring of NTBC-treated AKU patients is essential to avoid ocular and neurological sequelae. Therefore, raising awareness among different specialties is crucial for early detection of the disease, allowing for timely initiation of treatment and prevention of complications.
